# Greater Omental Torsion With Reactive Periappendicitis: A Case Report From a Second-Level Care Hospital

**DOI:** 10.7759/cureus.114502

**Published:** 2026-08-13

**Authors:** Alejandro Herbert Oliva Arvizu, Luis Donaldo García Romero, Luis Eduardo Nava Mata, Joseph Iván Benítez Jiménez, Cristian Jair Morales Manzo

**Affiliations:** 1 General Surgery, Hospital General Regional No. 1, Instituto Mexicano del Seguro Social (IMSS), Querétaro, MEX; 2 Surgery, Hospital General Regional No. 1, Instituto Mexicano del Seguro Social (IMSS), Querétaro, MEX; 3 Surgery, Instituto Mexicano del Seguro Social (IMSS), Mexico City, MEX

**Keywords:** acute abdomen, omental torsion, omentectomy, surgical case report, torsion of the greater omentum

## Abstract

Greater omental torsion is an uncommon cause of acute abdomen that frequently mimics more prevalent surgical pathologies such as acute appendicitis, making preoperative diagnosis challenging. We present the case of a 34-year-old overweight man (BMI: 27.7 kg/m²) who presented with a one-week history of right lower quadrant abdominal pain exacerbated by physical exertion and accompanied by fever reaching 38.5°C. Laboratory studies showed mild leukocytosis, and appendiceal ultrasonography did not demonstrate findings consistent with acute appendicitis, prompting further evaluation with computed tomography (CT) due to persistent symptoms and suspicion of an alternative intra-abdominal pathology. Non-contrast abdominopelvic computed tomography (CT) demonstrated a characteristic whirl sign suggestive of omental torsion, along with peripheral inflammatory changes. Exploratory laparotomy confirmed the complete ischemic torsion of the greater omentum and reactive periappendicitis. Total omentectomy and appendectomy were performed. The postoperative course was complicated by transient ileus, which resolved with conservative management. Histopathological examination revealed acute-on-chronic omental inflammation with fat necrosis and periappendicitis. Greater omental torsion should be considered in the differential diagnosis of right lower quadrant pain, particularly in overweight male patients. CT plays a fundamental role in preoperative identification, with the "whirl sign" being a key imaging finding. Timely recognition and appropriate management are associated with favorable clinical outcomes.

## Introduction

Omental torsion is a rare condition in which a mobile omental pedicle twists around a fixed point along its longitudinal axis, compromising its vascular supply and potentially mimicking other common causes of acute abdomen, particularly acute appendicitis [1,2]. Eitel first described this entity in 1899 [1]; however, Bush is credited with reporting the first case of segmental infarction in 1896 [2,3]. Omental torsion accounts for less than 1.1% of acute abdomen cases and is identified in approximately one out of every 600-800 appendectomies, with many cases diagnosed incidentally during surgery [1,2,4-6]. The vascular compromise initially affects venous drainage, leading to congestion and edema, and may subsequently impair arterial inflow, resulting in ischemia, hemorrhagic infarction, and necrosis. These changes contribute to the development of abdominal pain and local inflammation [2,4].

It typically occurs during the fourth and fifth decades of life, with a male predominance [2-9]; however, its incidence in the pediatric population has increased due to the rising prevalence of obesity in this age group [5,6]. The etiology of omental torsion has not been clearly established but may be classified as primary (in the absence of intra-abdominal pathology) or secondary (associated with hernias, tumors, cysts, postoperative scars, or adhesions) [3-6]. Multiple predisposing and precipitating factors have been described, including anatomical variants, obesity, increased size, weight, and the mobility of the omentum, which contribute to the greater frequency of right-sided torsion, as well as local trauma, occupational exposure to vibratory tools, hyperperistalsis after a heavy meal, sudden changes in body position, and increased intra-abdominal pressure [4-6].

Clinical presentation is usually nonspecific, with sudden-onset right lower quadrant abdominal pain being the most common symptom, frequently mimicking acute appendicitis [2-5]. Diagnostic adjuncts such as laboratory studies and abdominal ultrasonography often yield nonspecific findings; however, computed tomography (CT) has improved preoperative recognition by demonstrating characteristic findings, such as a whirling pattern of omental vessels and inflammatory changes within the omental fat [2-7]. Despite these advances, some cases are still diagnosed intraoperatively. Management may be conservative in selected uncomplicated cases, whereas surgical resection is indicated when symptoms persist or when ischemia, necrosis, or diagnostic uncertainty is present [2-8].

We present a case of omental torsion with reactive periappendicitis managed in a second-level care hospital through exploratory laparotomy, highlighting the preoperative and intraoperative findings.

## Case presentation

A 34-year-old overweight man (BMI: 27.7 kg/m²) with a sedentary lifestyle and no significant past medical history presented to the emergency department with a one-week history of colicky abdominal pain localized to the right iliac fossa, without radiation, exacerbated by physical exertion. Three days prior to admission, he developed fever up to 38.5°C and self-medicated with omeprazole, metamizole sodium, and a single dose of acetaminophen, achieving partial symptom relief. The day before admission, pain acutely worsened following the lifting of an approximately 15 kg object.

On admission, vital signs were as follows: blood pressure, 108/73 mmHg; heart rate, 80 beats per minute; respiratory rate, 19 breaths per minute; and body temperature, 36.8°C. The patient was alert and neurologically intact. Abdominal examination revealed a distended but soft abdomen with decreased bowel sounds, no signs of peritoneal irritation, and moderate tenderness on deep palpation of the lower abdomen, predominantly in the right iliac fossa. McBurney's sign was equivocal, while the remaining appendiceal signs were negative.

Laboratory investigations revealed leukocytosis of 12.4 × 10³/µL with neutrophilia (83.4%). Complete blood count, renal function tests, liver function tests, and urinalysis were otherwise unremarkable. Lipase was within normal laboratory limits (222 U/L), with no clinical or tomographic evidence of acute pancreatitis (Table 1).

**Table 1:** Laboratory Parameters at Admission Laboratory evaluation at admission revealed mild leukocytosis with neutrophilic predominance and a discrete increase in total bilirubin levels; all other hematologic indices, serum biochemical parameters, and urinalysis findings were within reference ranges ALT, alanine aminotransferase; AST, aspartate aminotransferase; LDH, lactate dehydrogenase; HPF, high-power field

Parameter	Result	Units	Reference Range
White blood cells	12.4	×10³/µL	4.0-11.0
Neutrophils	83.4	%	37.0-75.0
Hemoglobin	13	g/dL	11.0-18.0
Hematocrit	41.1	%	36.0-56.0
Platelets	373	×10³/µL	150-450
Glucose	103	mg/dL	74.0-106.0
Urea	34.2	mg/dL	20.0-43.0
Creatinine	0.8	mg/dL	0.50-1.10
Total bilirubin	1.9	mg/dL	0.30-1.20
Direct bilirubin	0.8	mg/dL	-
Indirect bilirubin	1.1	mg/dL	-
ALT	30	U/L	0.0-55.0
AST	30	U/L	5.00-34.0
Alkaline phosphatase	119	U/L	45.0-129.0
LDH	211	U/L	125.0-220.0
Amylase	46	U/L	30.0-110.0
Lipase	222	U/L	23.0-300.0
Leukocyte esterase	Negative	-	Negative
Nitrites	Negative	-	Negative
Urinary leukocytes	Negative	per HPF	Negative
Urinary bacteria	Negative	-	Negative

**Figure 1:**
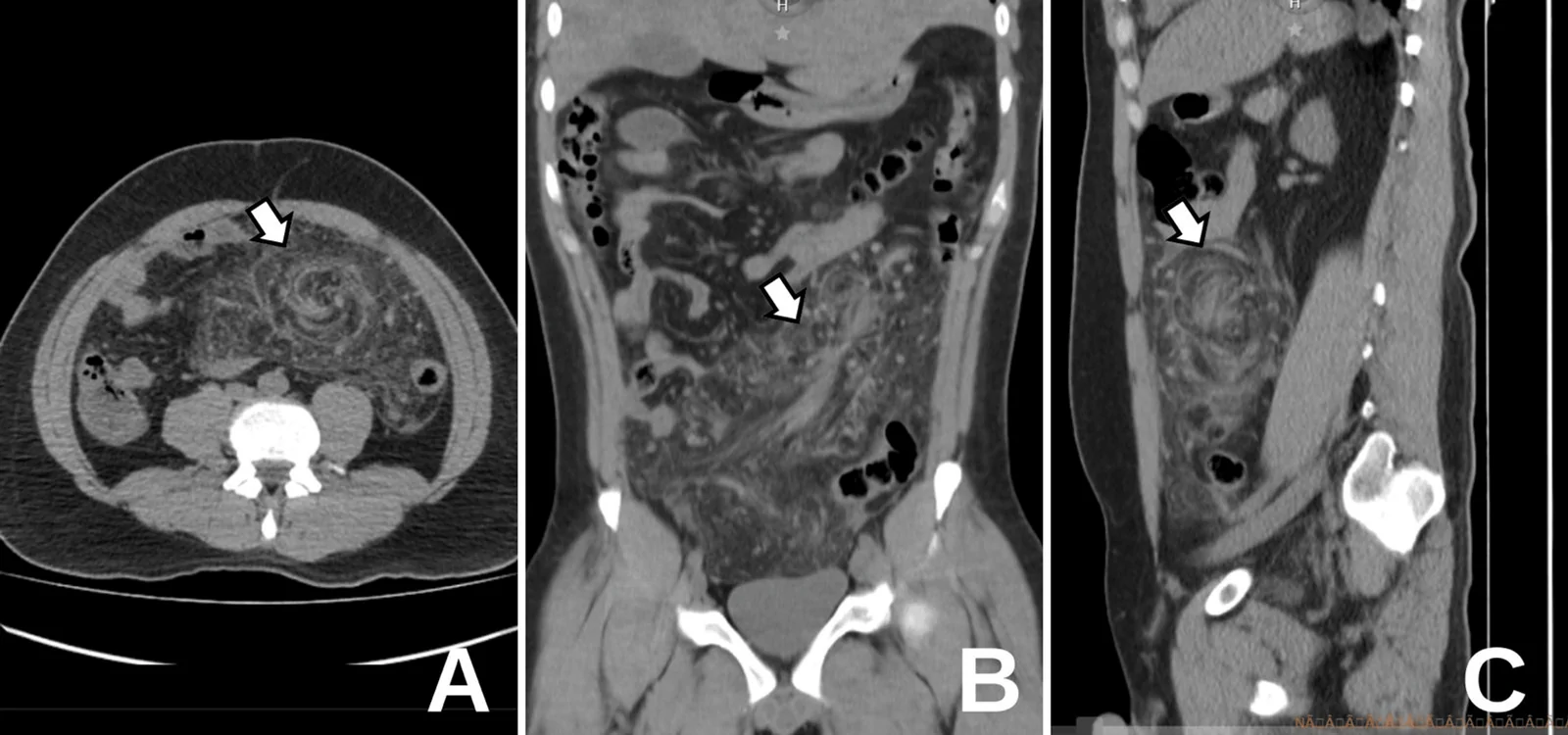
Abdominopelvic Computed Tomography in Non-contrast Slices Abdominal computed tomography in axial non-contrast slices (A), coronal reconstruction (B), and sagittal reconstruction (C) demonstrating increased density of the omental fat with a concentric and helical stranding pattern, forming the "whirl sign." Thickened vascular structures are observed converging toward a central point corresponding to the twisted vascular pedicle, associated with inflammatory changes in the adjacent adipose tissue. Findings are compatible with torsion of the greater omentum

Appendiceal ultrasonography did not demonstrate findings consistent with acute appendicitis. Due to diagnostic uncertainty, non-contrast abdominopelvic CT was performed, demonstrating a whirl-like appearance of the mesenteric fat and vessels rotating clockwise, consistent with omental torsion, along with peripheral inflammatory changes and minimal fluid in both paracolic gutters (Figure 1).

**Figure 2:**
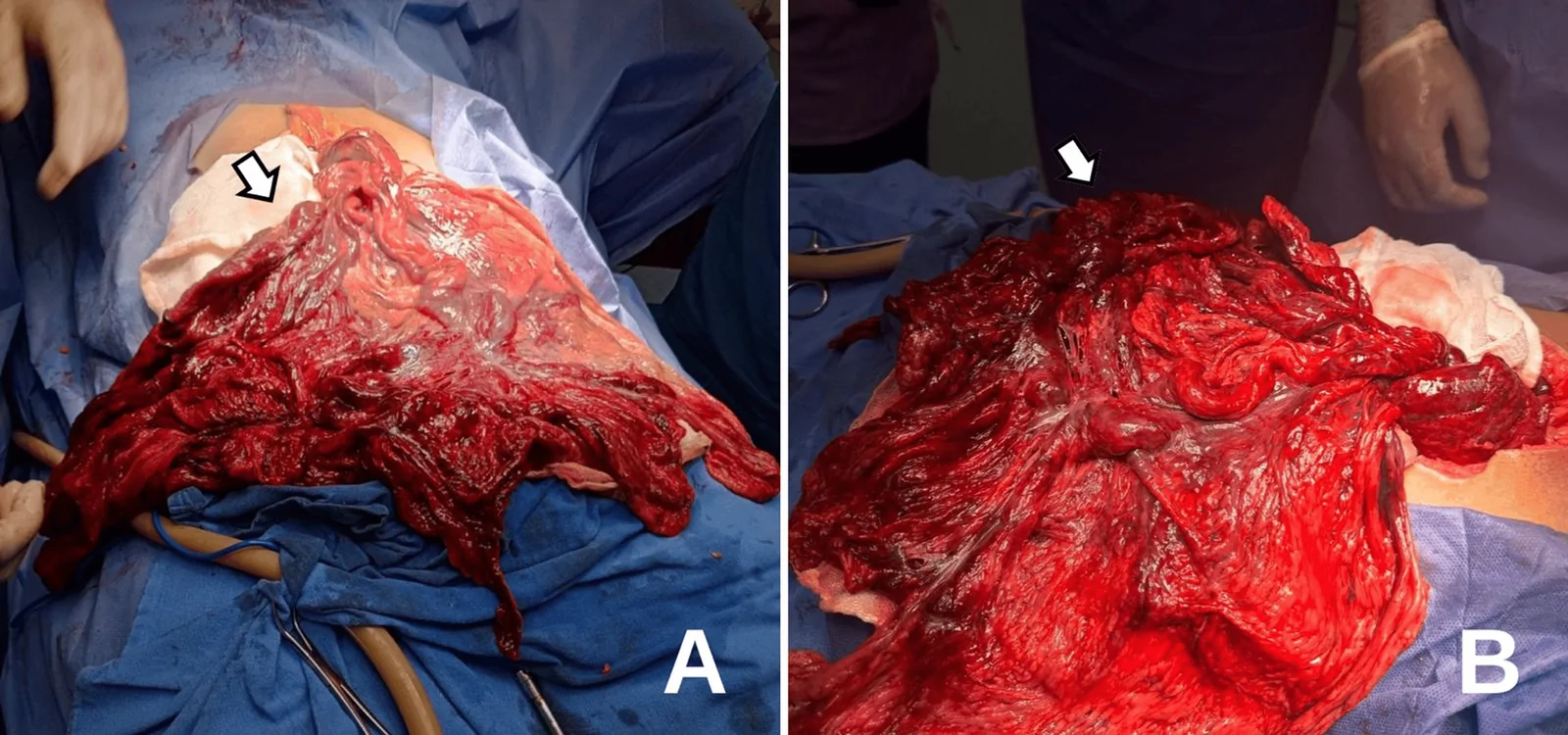
Intraoperative Findings Intraoperative findings during exploratory laparotomy demonstrating torsion of the greater omentum along its longitudinal axis (A), with marked ischemic and inflammatory changes and extensive hemorrhagic necrosis secondary to vascular compromise (B). The adjacent intestinal loops remained intact without evidence of macroscopic ischemic involvement

Based on these findings, exploratory laparotomy was performed, revealing torsion of the greater omentum with complete ischemia (Figure 2), associated with periappendiceal inflammatory changes and periappendiceal fluid. Total omentectomy and appendectomy were performed. The immediate postoperative course was initially uneventful.

**Figure 3:**
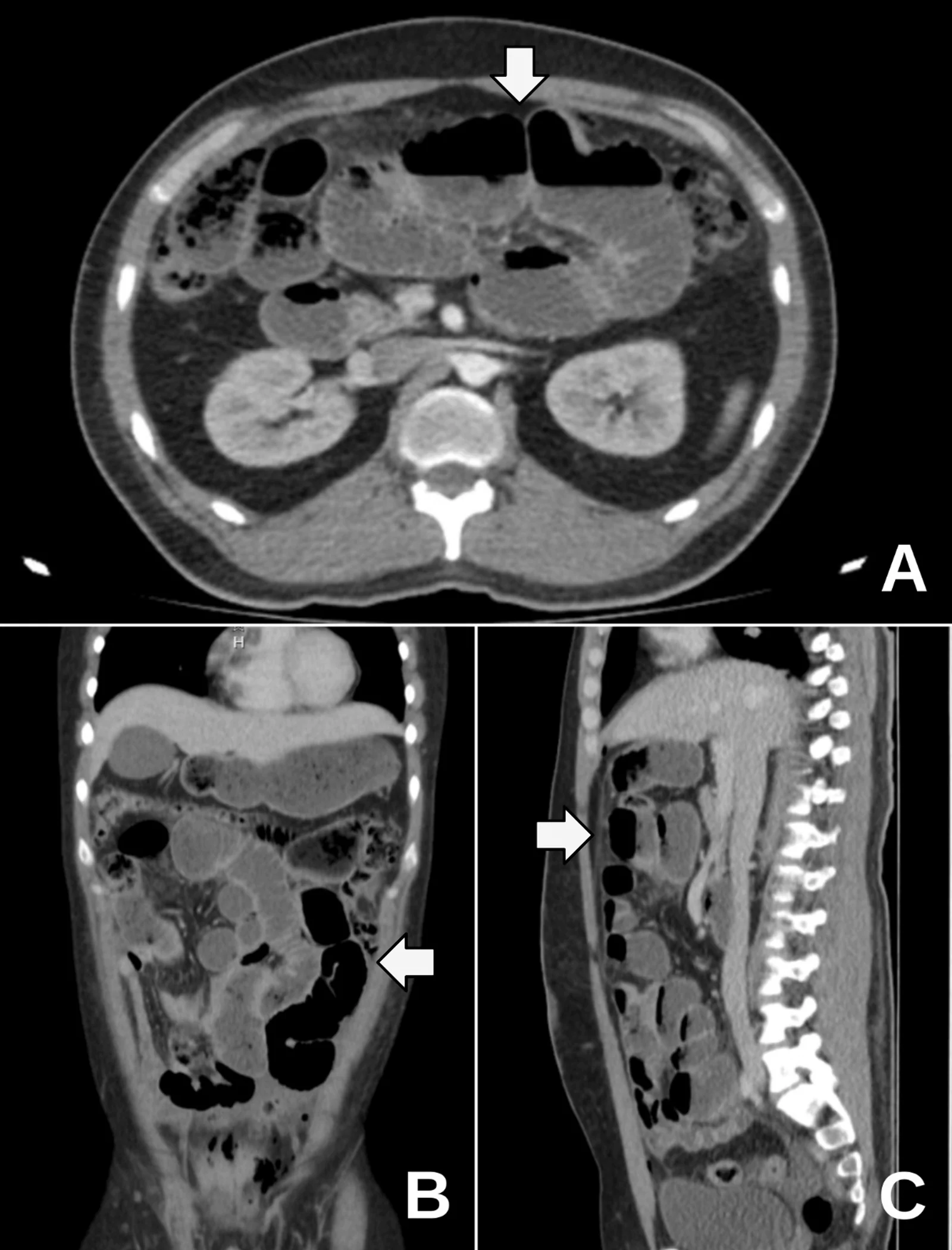
Follow-Up Contrast-Enhanced Thoracoabdominal CT Demonstrating Postoperative Paralytic Ileus Contrast-enhanced computed tomography (CT) in axial section (A) and coronal (B) and sagittal (C) reconstructions demonstrating diffuse dilation of small bowel loops with the predominance of fluid content and multiple air-fluid levels, without the identification of a definite transition point. These findings are consistent with paralytic ileus

On postoperative day 3, the patient developed fever, chills, malaise, and generalized colicky abdominal pain. Follow-up contrast-enhanced thoracoabdominal CT demonstrated diffuse dilation of small bowel loops with multiple air-fluid levels and no identifiable transition point, consistent with postoperative paralytic ileus (Figure 3). Conservative management with nasogastric decompression and antibiotic therapy led to resolution. The patient was discharged on postoperative day 7 in good clinical condition.

**Figure 4:**
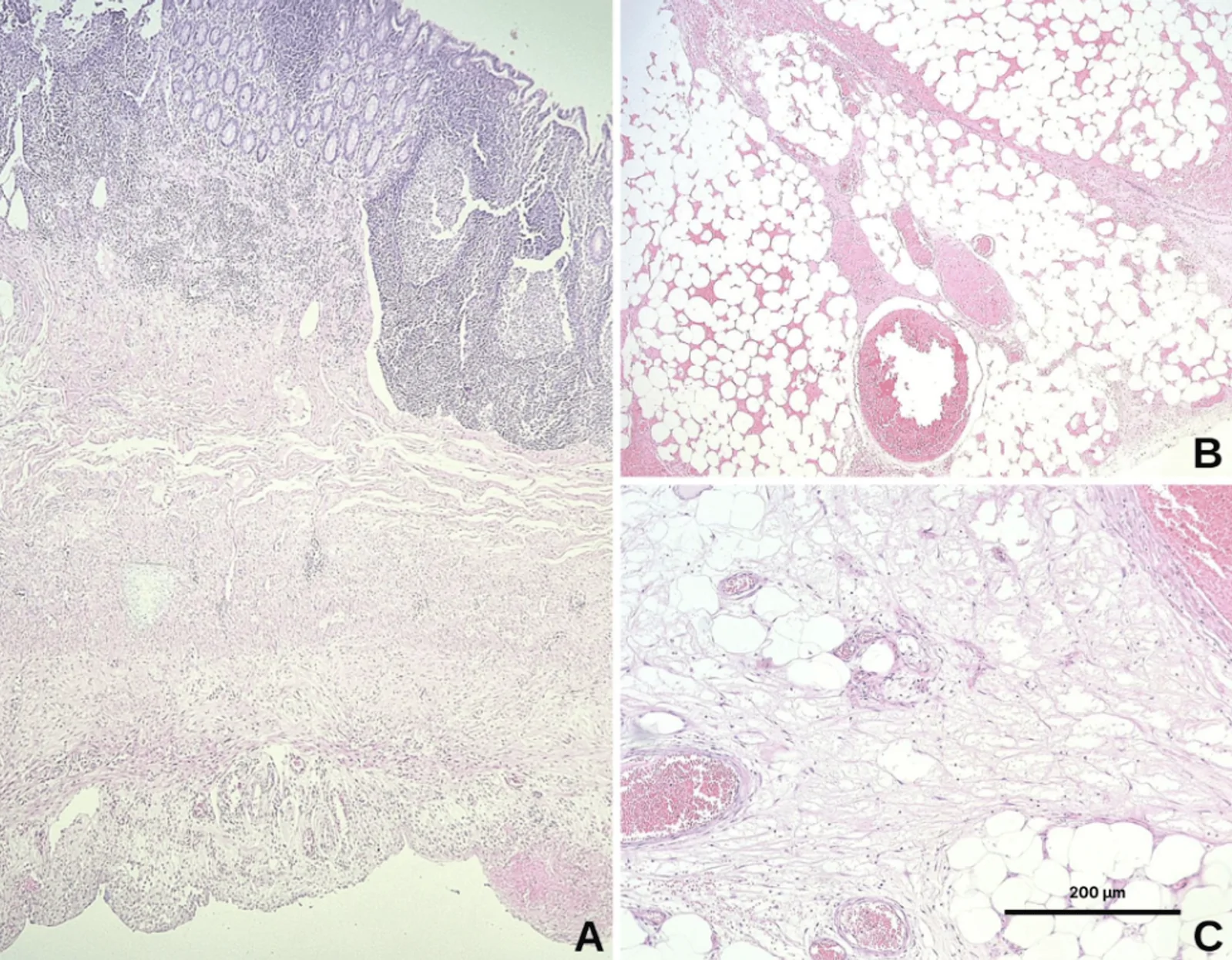
Histopathological Findings of the Appendix and Greater Omentum Histopathological examination of the cecal appendix and greater omentum stained with hematoxylin and eosin (H&E), obtained using a 10× objective (total magnification: 100×). The appendix (A) shows acute inflammatory infiltrate predominantly involving the serosa and periappendiceal adipose tissue with the preservation of the mucosa and muscularis propria, consistent with reactive periappendicitis. The greater omentum (B-C) demonstrates vascular congestion and hemorrhagic infarction with erythrocyte extravasation resulting in the ischemic necrosis of adipocytes. Scale bar = 200 µm. All images were obtained at the same magnification

Histopathological examination revealed acute-on-chronic inflammation of the greater omentum with hemorrhagic infarction and ischemic fat necrosis, as well as acute-on-chronic periappendicitis (Figure 4).

## Discussion

Greater omental torsion is an infrequent but clinically relevant cause of acute abdomen that may simulate more common surgical conditions such as acute appendicitis, as occurred in this case. It represents less than 1.1% of acute abdomen cases and is found in approximately one out of every 600-800 appendectomies; in many cases, diagnosis is established incidentally during surgery [1,2,4-6].

From a pathophysiological standpoint, omental torsion occurs when the greater omentum rotates around a central axis or pivot point, generally clockwise. This rotation compresses the tortuous omental veins, which collapse more easily than arteries, compromising venous return. Consequently, the distal segment becomes congested and edematous and may develop hemorrhagic extravasation, producing serosanguineous fluid in the peritoneal cavity. If torsion progresses, arterial flow, commonly involving the distal right epiploic artery, may also become occluded, leading to acute hemorrhagic infarction and eventual omental necrosis [2,4].

Up to 90% of cases present with right lower quadrant pain mimicking acute appendicitis [2,4,7]. Although nausea, vomiting, and fever may occur, they are usually less intense, with fever rarely exceeding 38°C [2-4,9,10]. However, in the present case, the patient developed a fever of 38.5°C, slightly exceeding the temperature range commonly described in the literature. The differential diagnosis of right lower quadrant abdominal pain includes several inflammatory and surgical conditions that may present with similar clinical features, including epiploic appendagitis, diverticulitis, Meckel's diverticulitis, and other localized intra-abdominal inflammatory processes [3,4,8]. Due to overlapping clinical manifestations, distinguishing these entities based solely on clinical evaluation can be challenging, emphasizing the importance of imaging studies, particularly computed tomography, for accurate diagnosis [3,4,9].

Anatomically, torsion occurs more frequently on the right side of the abdomen due to the greater length, weight, and mobility of this portion of the omentum, contributing to diagnostic confusion with acute appendicitis [2,4,5,8,11].

Overweight and obesity are significant predisposing factors, as increased omental fat mass favors torsion [2-6,11,12]. Acute physical exertion, as observed in this case, is consistent with previously described precipitating factors related to sudden increases in intra-abdominal pressure [4,11,12].

Computed tomography (CT) is the diagnostic modality of choice. Typical findings include a fatty omental mass with internal stranding and the displacement of adjacent structures. The "whirl sign," representing spiraled omental vessels, is a characteristic imaging feature. In advanced stages, free fluid and fat necrosis may be observed [2-5,8-10]. The clinical and radiological findings in this case are consistent with previously reported presentations of greater omental torsion, particularly the predominance of right lower quadrant pain, the association with overweight or obesity, and the characteristic whirl sign on CT [2-5,7-10]. Similar to other reported cases, the clinical presentation initially raised suspicion of acute appendicitis. However, the combination of complete ischemic torsion of the greater omentum and reactive periappendicitis represents an uncommon finding that may further contribute to diagnostic uncertainty.

The surgical resection of the necrotic omental segment remains the cornerstone of treatment in patients with persistent symptoms, suspected ischemia or necrosis, or diagnostic uncertainty [2-8]. In this case, exploratory laparotomy was performed because of complete ischemic torsion of the greater omentum. Total omentectomy and appendectomy were performed because of the associated periappendiceal inflammatory changes and reactive periappendicitis [7,10].

It is important to distinguish reactive periappendicitis from primary acute appendicitis. In the present case, histopathological examination demonstrated reactive periappendicitis without evidence of primary acute appendicitis, supporting the interpretation that the periappendiceal inflammatory changes were secondary to the adjacent omental inflammatory process. This distinction is clinically relevant because the macroscopic appearance of the appendix may suggest acute appendicitis in the setting of an inflamed right lower quadrant, whereas histopathological examination can establish whether the appendix represents the primary source of inflammation or a reactive finding.

The management of greater omental torsion may be either conservative or surgical, depending on the severity of symptoms, clinical stability, and the presence of ischemia or necrosis. Conservative management has been described in selected patients with mild, self-limited symptoms and no evidence of complications, allowing spontaneous resolution with close clinical observation. However, persistent or worsening symptoms, suspected ischemia or necrosis, or diagnostic uncertainty generally favors surgical exploration [3,4,8,9]. When surgery is indicated, both laparoscopic and open approaches have been described. Laparoscopic management offers the advantages of minimally invasive surgery, including reduced postoperative pain, shorter hospital stay, faster recovery, and the direct visualization of the abdominal cavity while also allowing the resection of the affected omentum. Its limitations include the need for appropriate expertise and equipment and potential technical difficulties in cases of extensive necrosis or severe inflammatory changes. Open surgery provides rapid and direct access to the abdominal cavity and may be preferable when extensive omental necrosis, severe inflammation, or diagnostic uncertainty requires prompt exploration, although it is associated with greater surgical trauma and longer postoperative recovery [3,4,9,12]. In the present case, exploratory laparotomy was selected because of the suspected acute surgical abdomen and the need for definitive diagnosis and treatment of complete ischemic torsion of the greater omentum.

Postoperative ileus, although uncommon, has been reported following extensive omental resection and resolved conservatively in this patient [13].

## Conclusions

Greater omental torsion remains a diagnostic challenge and represents an uncommon etiology of acute abdomen due to its nonspecific clinical presentation and resemblance to more common inflammatory intra-abdominal processes. Predisposing factors such as overweight and obesity increase clinical suspicion. Abdominopelvic CT is the diagnostic tool of choice, with the "whirl sign" serving as a key imaging feature for preoperative identification.

Despite advances in imaging, diagnosis is frequently confirmed intraoperatively. Surgical resection remains the preferred treatment for patients with persistent symptoms, ischemia, necrosis, or diagnostic uncertainty, although conservative management may be considered in selected uncomplicated cases. The early detection and appropriate interpretation of imaging findings are essential to optimize therapeutic management and reduce morbidity.
